# Radiomics-Guided Multi-Sequence Learning for Pathological Complete Response Prediction from Breast MRI with Missing Auxiliary Sequences

**DOI:** 10.3390/jimaging12060271

**Published:** 2026-06-18

**Authors:** Xinyuan Xiang, Wenyu Yin, Jiayue Li

**Affiliations:** 1 School of Information and Communication Engineering, Beijing University of Posts and Telecommunications, Beijing 100876, China; xiangxinyuan@bupt.edu.cn (X.X.); yinwenyu@bupt.edu.cn (W.Y.); 2 School of Artificial Intelligence and Computer Science, North China University of Technology, Beijing 100144, China

**Keywords:** breast cancer, pCR prediction, multi-sequence MRI, radiomics, missing sequences

## Abstract

Pathological complete response (pCR) after neoadjuvant chemotherapy (NACT) provides an endpoint for treatment evaluation in breast cancer. Multi-sequence breast MRI can support pCR prediction, but routine examinations may lack usable T1-weighted or T2-weighted sequences. Many models merge radiomic and deep features by concatenation, leaving the interaction between handcrafted descriptors and learned representations weakly specified. We developed a radiomics-guided framework for pCR prediction from multi-sequence breast MRI. The model uses a multi-branch 2.5D encoder for sequence-specific features, radiomics-guided channel recalibration, and masked token fusion to aggregate available sequence tokens. We evaluated the framework on 157 patients from the I-SPY1 Trial cohort with patient-level five-fold cross-validation, fixed sequence-combination analysis, and slice-window sensitivity analysis. The full model achieved 78.4% accuracy and 0.809 AUC, compared with 75.8% accuracy and 0.788 AUC for the strongest channel-concatenation baseline. In this cohort, radiomics-guided multi-sequence learning was feasible, with external validation required before clinical interpretation.

## 1. Introduction

Breast cancer is one of the most common malignancies among women worldwide. Neoadjuvant chemotherapy (NACT) reduces tumor burden before surgery in patients with locally advanced or biologically aggressive disease and gives clinicians an early view of the treatment response [1]. Pathological complete response (pCR) serves as a clinically meaningful endpoint for evaluating the treatment effect [2]. Preoperative pCR prediction can inform treatment planning and response monitoring.

MRI supports breast cancer diagnosis and treatment evaluation through high soft-tissue contrast and multi-parametric acquisition. Breast MRI can assess residual disease and pathological responses after NACT [3]. Dynamic contrast-enhanced (DCE) MRI, T1-weighted imaging, and T2-weighted imaging capture tumor morphology, vascularity, internal heterogeneity, and surrounding tissue characteristics. Recent studies have used quantitative breast MRI analysis for treatment response prediction [4,5,6,7,8].

Radiomics converts lesion shape, intensity distribution, and texture heterogeneity into handcrafted quantitative descriptors. These features add interpretable information beyond visual assessment [9,10,11]. Radiomics-based and radiomics-assisted models have shown value in breast cancer response prediction [6,7,8], which suggests that handcrafted descriptors and learned image representations may capture partly different information.

Multi-sequence pCR prediction faces two practical constraints. Many models assume that all MRI sequences are available during training and inference. Clinical breast MRI does not always satisfy this assumption: T1-weighted or T2-weighted series may be missing or unusable because of protocol differences, scan time constraints, motion artifacts, or sequence-specific quality failures. Contrast-enhanced imaging is central to lesion characterization, but ancillary sequences vary more across examinations. Missing-modality methods in other imaging tasks offer useful precedents. HeMIS trains one model for any subset of available sequences by aggregating sequence-specific statistics [12]. Modality dropout removes input modalities during training to improve robustness under missing inputs [13]. Cross-modal knowledge distillation transfers information from a full-modality teacher to a partial-modality student [14]. Transformer-based methods such as mmFormer and ShaSpec [15,16] combine shared and modality-specific representations through attention, and uncertainty-aware fusion weights modalities according to estimated reliability [17]. Much of this work focuses on brain tumor segmentation with four MRI sequences, with less attention to radiomics-guided pCR classification.

Radiomics integration adds another constraint. Many breast MRI studies combine radiomic and deep features as parallel streams, often by concatenating features before the classifier head [6,7,8]. Attention or recalibration may appear at the fused-feature stage, but the radiomic descriptors usually remain a separate evidence stream. We use radiomic descriptors as a conditioning input for sequence-specific deep encoders. The gating module produces channel-wise scaling factors before cross-sequence fusion, so radiomics modulates deep representations without adding another feature block.

We developed RGM-Net for pCR prediction from multi-sequence breast MRI. The model extracts sequence-specific features from contrast-enhanced, T1-weighted, and T2-weighted images with a multi-branch 2.5D encoder. A radiomics-guided gating module recalibrates deep features with handcrafted radiomic descriptors, and masked token fusion aggregates the sequence tokens present under fixed sequence-availability settings. The main contributions are:

- A radiomics-guided pCR prediction framework that integrates handcrafted radiomic descriptors with sequence-specific deep features through channel-wise modulation.
- A gating module that maps radiomic features to channel-wise scaling factors for each MRI sequence encoder.
- A masked token fusion strategy for fixed sequence-availability settings without imputing unavailable sequences.

We evaluated the framework on the I-SPY1 Trial cohort using patient-level five-fold cross-validation.

## 2. Methodology

### 2.1. Overview

**Figure 1:**
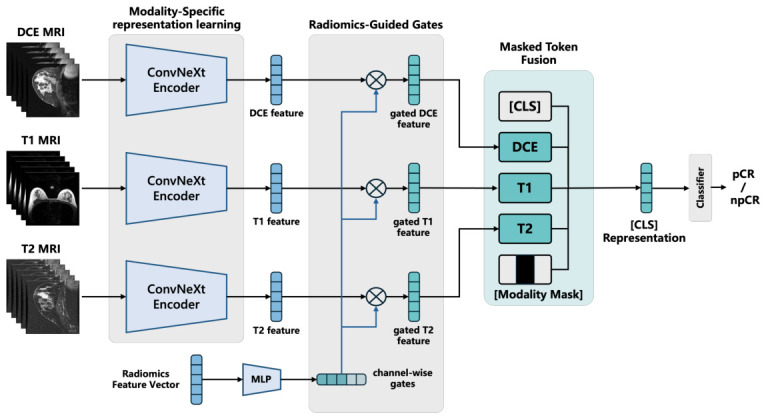
Overall framework of the proposed RGM-Net.

Figure 1 shows the overall RGM-Net framework for pCR prediction from multi-sequence breast MRI with potentially missing auxiliary sequences. Let $x=\left\{X^{\left(m\right)}\right\},\;m\in \left[1,2,3\right]$ denote the available MRI sequences, where $X^{\left(m\right)}$ is the input image of the *m*-th sequence. We considered DCE MRI, T1-weighted imaging, and T2-weighted imaging. The lesion region provided a radiomics feature vector *R* as handcrafted side information.

RGM-Net contains a multi-branch 2.5D feature encoder, a radiomics-guided gating (RGG) module, and a masked token fusion (MTF) module. Independent branches extract sequence-specific deep features. The RGG module converts radiomics features into channel-wise weights and recalibrates the deep representations. The MTF module then fuses the available sequence tokens and excludes missing sequences from attention. A prediction head maps the fused representation to the pCR probability.

### 2.2. Multi-Branch Feature Encoding

Multi-sequence breast MRI contains heterogeneous information across imaging sequences. To preserve sequence-specific characteristics, each sequence enters a dedicated branch with the same network architecture but independent parameters. Each branch uses a 2.5D ConvNeXt backbone as its feature extractor.

For the *m*-th sequence, the default configuration stacks five adjacent slices centered on the lesion along the channel dimension to form a 2.5D input. The 2.5D format adds through-plane context compared with single-slice 2D analysis while avoiding the computational cost and data demand of full 3D convolution. Given the input $X^{\left(m\right)}\in R^{C\times H\times W}$, the encoder produces the deep feature map $F^{\left(m\right)}$:

(1) $$F^{\left(m\right)}=E^{\left(m\right)}\left(X^{\left(m\right)}\right),$$

where $E^{\left(m\right)}\left(\cdot \right)$ denotes the ConvNeXt encoder for the *m*-th sequence. Separate branches allow the model to learn representations matched to DCE, T1-weighted, and T2-weighted MRI.

### 2.3. Radiomics-Guided Gating

Deep convolutional features capture hierarchical image patterns, but the training objective does not force them to preserve clinically defined quantitative characteristics. Radiomics features describe lesion morphology, intensity distribution, and textural heterogeneity with fixed handcrafted measurements. The RGG module uses these descriptors to modulate deep feature channels.

Given the radiomics feature vector *R*, a multi-layer perceptron (MLP) projects it into a channel-wise gating vector:

(2) $$G=\sigma \left(\mathrm{MLP}(R)\right),$$

where *G* denotes the learned gating weights and σ(·) denotes the sigmoid function. The sigmoid constrains the output to [0,1]. The gating vector scales the encoded deep feature map channel by channel:

(3) $${\tilde{F}}^{\left(m\right)}=G⊙F^{\left(m\right)},$$

where ⊙ denotes element-wise multiplication, and ${\tilde{F}}^{\left(m\right)}$ is the radiomics-modulated feature map of the *m*-th sequence.

The gating formulation treats radiomics as a recalibration signal rather than an additional feature source. Radiomic descriptors provide deterministic summaries of lesion shape, intensity, and texture on the same region used for imaging analysis. Channel-wise gating supplies these summaries as a bounded prior that can attenuate or retain existing deep channels. Radiomic descriptors and deep features may overlap. Additive fusion would treat them as separate evidence sources and could double-count shared information. Multiplicative gating limits this risk by modulating features learned by the encoder, without assuming that the handcrafted and deep representations are independent.

### 2.4. Masked Token Fusion

MTF supports inference when one or more auxiliary sequences are missing. For each sequence *m*, the model globally pools the radiomics-modulated feature map ${\tilde{F}}^{\left(m\right)}$ and projects it into a sequence token $T^{\left(m\right)}\in R^{128}$:

(4) $$T^{\left(m\right)}=P\left({\tilde{F}}^{\left(m\right)}\right),$$

where P(·) denotes global average pooling followed by a linear layer. A learnable [CLS] token is prepended to the sequence tokens for global representation aggregation. The availability mask $M^{\left(m\right)}\in \left\{0,1\right\}$ indicates whether sequence *m* is present. Mask-aware attention then fuses the projected tokens:

(5) $$\hat{T}=\mathrm{Attention}\left(T^{\left(1\right)},T^{\left(2\right)},T^{\left(3\right)};M\right).$$

In the attention computation, the mask assigns −∞ to logits from missing sequences ($M^{\left(m\right)}=0$). The softmax then gives these positions zero attention weight. The [CLS] token aggregates information from present sequence tokens only.

Layer Normalization and a linear classifier transform the fused global representation into the final pCR prediction. In the sequence-availability experiments, unavailable sequence tokens are excluded by the mask rather than imputed; the evaluated sequence combinations are detailed in the experimental section.

The masking step follows the standard transformer convention of assigning −∞ to attention logits at masked positions. In RGM-Net, this step sits after radiomics-guided gating and before the classification head. The main comparison uses all three sequences for each image-based model. The sequence-availability analysis excludes unavailable sequences according to each fixed setting. A single fusion module handles full-sequence and partial-sequence inputs, without imputation, modality-specific submodels, or retraining for each sequence combination.

### 2.5. Loss Function

The training objective combines cross-entropy loss with focal loss. Cross-entropy provides standard supervision, and focal loss down-weights easy examples to reduce the influence of class imbalance. The two losses are

(6) $$L_{\mathrm{ce}}=-\left[y\log(p)+(1-y)\log(1-p)\right],$$

(7) $$L_{\mathrm{focal}}=-\alpha {\left(1-p_{t}\right)}^{\gamma }\log\left(p_{t}\right),$$

where *y* is the ground-truth label, *p* is the predicted probability for the positive class, and $p_{t}=p$ for positive samples and $p_{t}=1-p$ for negative samples. α and γ denote the weighting and focusing parameters, respectively. The total loss is

(8) $$L=L_{\mathrm{ce}}+L_{\mathrm{focal}}.$$

## 3. Experiments and Results

### 3.1. Data Acquisition and Preprocessing

#### 3.1.1. Dataset and Cohort

The experiments used the I-SPY1/ACRIN 6657 multi-center NACT cohort from The Cancer Imaging Archive (TCIA) [18,19]. Contrast-enhanced images, structural tumor volume (STV) masks, patient-level pCR labels, and standardized radiomic features came from the ISPY1-Tumor-SEG-Radiomics analysis-result dataset [20,21]. The data providers had converted the baseline pre-treatment DCE-MRI volumes to NIfTI, applied bias correction, resampled them to $1\;{\mathrm{mm}}^{3}$ isotropic resolution, and z-score-normalized the images. The analysis-result dataset did not include T1-weighted or T2-weighted images, so we retrieved the corresponding baseline DICOM series from the original I-SPY1 collection using acquisition timepoint metadata, series descriptions, and protocol fields. We excluded patients without reliably identified baseline T1-weighted or T2-weighted series. The final cohort included 157 patients, with 43 pCR and 114 non-pCR cases.

#### 3.1.2. Image Preprocessing and Input Construction

The first post-contrast DCE volume served as the contrast-enhanced input, and the expert-confirmed STV mask on this volume defined the lesion region of interest. We converted the retrieved T1-weighted and T2-weighted DICOM series to NIfTI, applied N4 bias-field correction, resampled them to $1\;{\mathrm{mm}}^{3}$ isotropic resolution, rigidly registered them to the corresponding pre-contrast DCE volume with mutual information, and normalized them with patient-level z-scores. After registration, all three sequences used the DCE-space STV mask.

Model inputs followed a 2.5D sampling strategy. For each patient, we identified slices intersecting the STV, stacked consecutive slices along the channel dimension, and resized each stack to 224×224 pixels. The main configuration used five adjacent slices per input; sensitivity experiments repeated the pipeline with three and seven adjacent slices. During inference, the model produced one pCR probability for each 2.5D input. The patient-level probability equaled the average of the top 20% slice-window probabilities, with at least one input retained per patient. We used a threshold of 0.5 for patient-level binary classification.

#### 3.1.3. Sequence Availability, Splitting, and Radiomics

In the main baseline comparison, all the image-based methods used all three MRI sequences, with no random sequence dropping. We evaluated additional fixed sequence-availability settings, including T1-weighted + T2-weighted, to characterize behavior under different sequence combinations. Patient-level stratified five-fold cross-validation kept all 2.5D inputs from the same patient within the same fold. In each fold, 80% of the patients formed the training set and 20% formed the test set.

Each patient had 370 CaPTk radiomic features distributed with the ISPY1-Tumor-SEG-Radiomics dataset [20,22]. Before input to the radiomics-guided gating module, each fold standardized the features with the mean and standard deviation from its training split.

### 3.2. Implementation Details

We implemented the framework in PyTorch 2.5.0. Each sequence-specific encoder used ConvNeXt-Tiny initialized with publicly available ImageNet-1k pretrained weights. The DCE, T1-weighted, and T2-weighted encoders shared the same architecture but had independent parameters. The radiomics-guided gating module used a two-layer MLP to map the 370-dimensional radiomic feature vector to a channel-wise gating vector matching the output channel dimension of the corresponding ConvNeXt-Tiny encoder. The MLP contained one hidden layer with 256 units, ReLU activation, a dropout of 0.3, and a final sigmoid activation that constrained the gating values to [0,1]. The masked token fusion module used one transformer encoder block with multi-head self-attention (4 heads, embedding dimension 128), a feed-forward sublayer, and Layer Normalization, and prepended a learnable [CLS] token to the sequence tokens for global representation aggregation.

The training used AdamW with an initial learning rate of $1\times 10^{-4}$ and weight decay of 0.05. We kept these ConvNeXt fine-tuning defaults fixed to avoid tuning hyperparameters on the small cohort. A cosine annealing scheduler decayed the learning rate. The model was trained for a fixed 400 epochs with a batch size of 32. No validation subset or test-fold information was used for checkpoint selection. The data augmentation included horizontal flipping and random rotation up to $\pm 15^{\circ }$. In fixed sequence-availability experiments, the model masked unavailable sequence tokens according to the evaluated setting. The slice-window sensitivity analysis repeated the same training and evaluation protocol with three, five, and seven adjacent slices per sequence input.

The combined loss summed the cross-entropy and focal loss terms with equal weight (λ=1); we did not use a learnable or fold-wise tuned weighting. The focal loss parameters were α=0.25 and γ=2.0, following the original focal loss formulation. Keeping these values fixed reduced fold-dependent hyperparameter choices for the 157-patient cohort. All experiments ran on a single NVIDIA RTX A6000 GPU.

### 3.3. Evaluation Metrics

We assessed classification performance with accuracy, sensitivity, specificity, precision, and F1-score:

(9) $$\mathrm{Accuracy}=\frac{TP+TN}{TP+TN+FP+FN},$$

(10) $$\mathrm{Sensitivity}=\frac{TP}{TP+FN},$$

(11) $$\mathrm{Specificity}=\frac{TN}{TN+FP},$$

(12) $$\mathrm{Precision}=\frac{TP}{TP+FP},$$

(13) $$F1\text{-}\mathrm{score}=\frac{2\times \mathrm{Precision}\times \mathrm{Sensitivity}}{\mathrm{Precision}+\mathrm{Sensitivity}},$$

(14) $$\mathrm{Balanced}\;\mathrm{Accuracy}=\frac{\mathrm{Sensitivity}+\mathrm{Specificity}}{2},$$

where TP, TN, FP, and FN denote true positives, true negatives, false positives, and false negatives, respectively. We also report the area under the ROC curve (AUC). Quantitative results are reported as mean ± standard deviation across the five cross-validation folds. Visual summaries show balanced accuracy and AUC with two-sided 95% confidence intervals estimated across folds.

### 3.4. Performance and Baseline Comparison

The main comparison used the same three-sequence input for all deep-learning baselines, with no random sequence dropping. For ResNet [23], ConvNeXt [24], EfficientNetV2 [25], and ViT [26], the DCE, T1-weighted, and T2-weighted 2.5D inputs were concatenated along the channel dimension and fed into a single backbone. This channel-concatenation baseline used the same image information as RGM-Net but omitted radiomics guidance and token-level masked fusion.

Table 1 reports the patient-level five-fold cross-validation results for all the main comparison methods.

**Figure 2:**
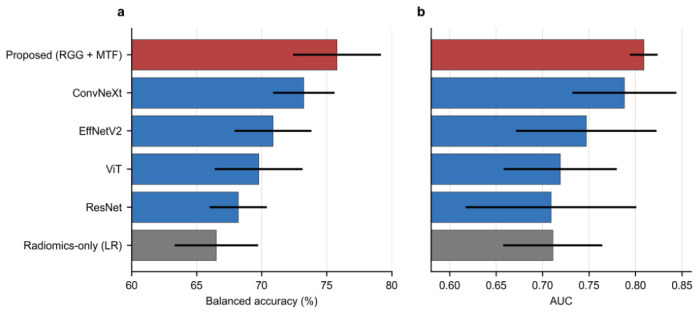
Main comparison under patient-level five-fold cross-validation. (**a**) Balanced accuracy and (**b**) AUC for the radiomics-only baseline, the channel-concatenation deep-learning baselines, and the proposed framework. Error bars indicate two-sided 95% confidence intervals estimated across the five folds.

Among the channel-concatenation baselines, ConvNeXt performed best (Acc 75.8 ± 1.2%, AUC 0.788 ± 0.045), followed by EffNetV2 (Acc 74.5 ± 1.7%, AUC 0.747 ± 0.061). The radiomics-only logistic regression baseline reached an AUC of 0.711 ± 0.043. RGM-Net gave the highest mean values across the evaluated metrics, with 78.4 ± 2.2% accuracy, 75.8% balanced accuracy, and 0.809 ± 0.012 AUC. Relative to ConvNeXt, RGM-Net improved the accuracy by 2.6 percentage points and AUC by 0.021. Figure 2 summarizes the balanced accuracy and AUC.

**Figure 3:**
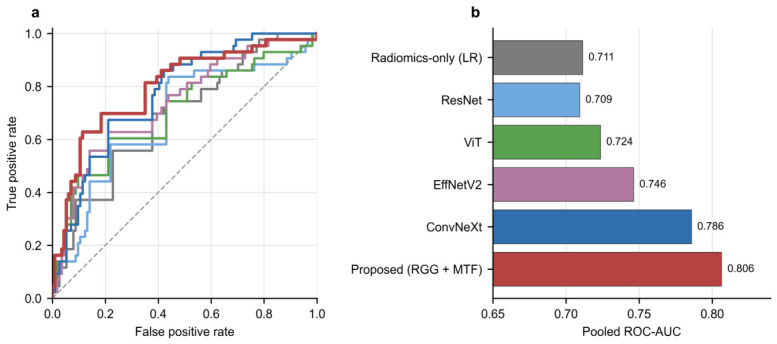
Pooled patient-level out-of-fold ROC analysis for the main comparison. Different colors denote different evaluated methods and are consistent between panels (**a**,**b**). (**a**) ROC curves generated from the patient-level prediction scores in each held-out fold. (**b**) Corresponding pooled ROC-AUC values. The fold-wise mean AUC values in Table 1 remain the primary quantitative comparison.

Pooled out-of-fold patient-level prediction scores gave a ROC-AUC of 0.806 for RGM-Net and 0.786 for ConvNeXt. Figure 3 shows the curves and pooled ROC-AUC values.

### 3.5. Ablation Study

The ablation study compared four configurations: (i) a ConvNeXt baseline using channel-concatenated DCE, T1-weighted, and T2-weighted inputs; (ii) the baseline with MTF only; (iii) the baseline with RGG only; and (iv) the full framework with both RGG and MTF. All configurations used the same patient-level five-fold cross-validation protocol. Table 2 gives the results.

Adding MTF alone increased the accuracy from 75.8% to 76.5%, while the mean AUC decreased from 0.788 to 0.771. Adding RGG alone produced the same mean accuracy of 76.5% and improved the sensitivity and F1-score, with a mean AUC of 0.765. The full model gave the best configuration (Acc 78.4 ± 2.2%, AUC 0.809 ± 0.012), corresponding to a 2.6-percentage-point accuracy increase and 0.021 AUC increase over the ConvNeXt baseline. The small cohort limits component-level conclusions from fold-mean point estimates.

### 3.6. Sequence Combination Analysis

The sequence-combination analysis covered four fixed availability settings: T1-weighted + T2-weighted, contrast-enhanced + T1-weighted, contrast-enhanced + T2-weighted, and all three sequences. Table 3 reports the results.

The T1-weighted + T2-weighted setting reached 73.9 ± 2.1% accuracy and 0.719 ± 0.044 AUC. DCE + T2 reached 75.2 ± 2.4% accuracy and 0.722 ± 0.079 AUC, whereas DCE + T1 reached 76.4 ± 1.4% accuracy and 0.800 ± 0.050 AUC. The three-sequence configuration had the highest mean values across all evaluated metrics.

### 3.7. Slice-Window Sensitivity Analysis

Because 2.5D modeling requires a fixed slice-window size, we tested three, five, and seven adjacent slices per sequence input. All settings used the same patient-level five-fold cross-validation protocol and the same top 20% slice-window aggregation strategy. Table 4 reports the results.

**Figure 4:**
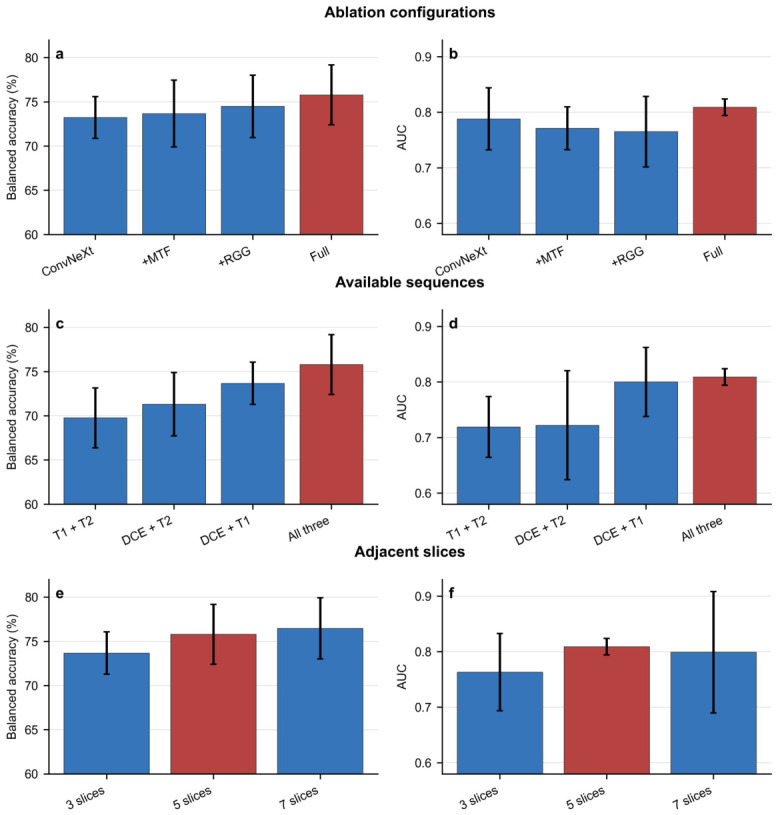
Summary of component ablation, fixed sequence-combination, and slice-window sensitivity analyses. (**a**,**c**,**e**) Balanced accuracy and (**b**,**d**,**f**) AUC across incremental model configurations, available sequence settings, and adjacent-slice windows, respectively. Error bars indicate two-sided 95% confidence intervals estimated across the five folds.

Three adjacent slices produced lower accuracy (76.4 ± 1.4%) and AUC (0.763 ± 0.056) than the default five-slice setting. Seven adjacent slices matched the five-slice mean accuracy and increased the sensitivity and F1-score, but the mean AUC decreased to 0.799 ± 0.088 with larger fold-to-fold variability. Five adjacent slices remained the default because they achieved the highest mean AUC and the most stable AUC among the tested slice windows. Figure 4 summarizes the ablation, sequence-combination, and slice-window analyses.

### 3.8. Visualization and Interpretability

Grad-CAM maps and attention-weight inspection provided qualitative checks of model behavior.

#### 3.8.1. Gradient-Based Class Activation Maps

**Figure 5:**
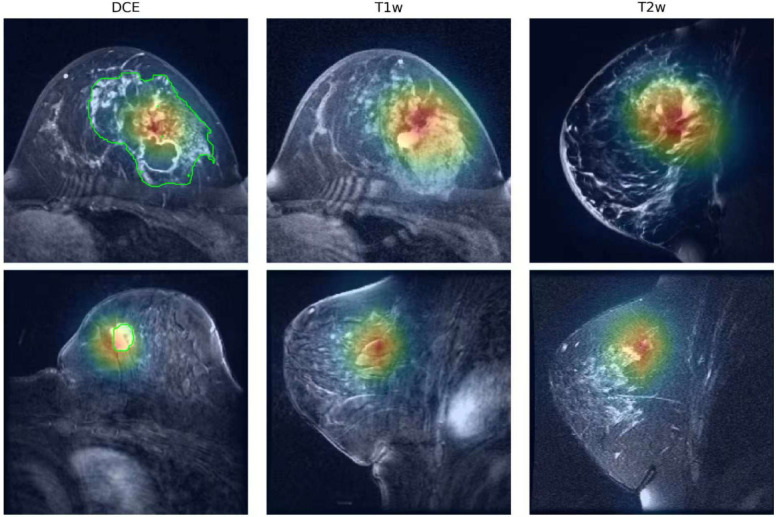
Representative Grad-CAM maps for the three sequence-specific encoders, shown for a correctly predicted pCR patient (top row) and a correctly predicted non-pCR patient (bottom row). For each patient, the central 2.5D input is shown alongside the corresponding Grad-CAM heatmaps for the DCE, T1-weighted, and T2-weighted branches. The green contours indicate the tumor boundaries.

For Grad-CAM, the target class was the predicted pCR class and the target layer was the final convolutional stage of each sequence-specific ConvNeXt-Tiny encoder. Figure 5 shows representative maps for a correctly predicted pCR patient and a correctly predicted non-pCR patient across the DCE, T1-weighted, and T2-weighted branches. The activation maps mainly focused on the lesion and surrounding regions.

#### 3.8.2. Attention Weights in the Masked Token Fusion Module

Attention-weight inspection focused on the [CLS] token and the available sequence tokens when all three sequences were present. The analysis is descriptive, rather than a causal explanation of sequence importance. The DCE token received the largest average attention weight (mean ≈0.51), followed by the T1-weighted token (≈0.27) and the T2-weighted token (≈0.22). When a fixed sequence-availability setting masked an auxiliary sequence, its attention contribution became zero by construction, and the attention mass shifted to the remaining sequence tokens without retraining.

## 4. Discussion

On the I-SPY1 Trial cohort, RGM-Net achieved a higher fold-mean accuracy, F1-score, and AUC than the channel-concatenation baselines. ConvNeXt was the strongest baseline, supporting its use as the image backbone. The fixed sequence-combination analysis favored the full three-sequence setting, while DCE + T1 outperformed T1 + T2 and DCE + T2 in AUC. Adding either RGG or MTF alone produced limited gains, but combining both modules yielded the best fold-mean performance. The slice-window analysis favored the default five-slice 2.5D input: seven slices increased the sensitivity and F1-score, but showed a lower mean AUC and larger fold-to-fold variability. The absolute margins remained modest.

The radiomics-guided gating module offers one way to connect handcrafted descriptors with deep features. Deep encoders learn spatially distributed patterns that are difficult to specify analytically. Radiomic descriptors provide fixed summaries of shape, intensity, and texture. RGG uses the descriptors as a side input that produces channel-wise scaling factors. This mechanism is best interpreted as feature recalibration. The present experiments do not show that radiomic descriptors and deep features provide independent evidence. Dedicated redundancy analysis would be needed to quantify overlap between the two representations.

Masked token fusion lets the model exclude unavailable sequence tokens from attention under fixed sequence-availability settings. In the main comparison, every image-based method used all three sequences; the sequence-availability analysis then tested fixed combinations without random sequence dropping. Clinical missingness in breast MRI may correlate with acquisition protocols, technical failures, and institutional practice. The present results should be limited to the fixed combinations evaluated here.

### Limitations

Several limitations affect the interpretation of the results. The evaluation used a single retrospective cohort of 157 patients from I-SPY1. Patient-level five-fold cross-validation reduced the dependence on one split, but the sample size remains small for a multi-branch network with token-level fusion. The risk of overfitting remains, and independent external validation was not performed.

The sequence-availability analysis covered a small set of fixed combinations. These settings support controlled comparison, but they may not reflect clinical missingness patterns, which can depend on acquisition protocols, patient factors, or imaging site. The model also did not include clinical variables such as treatment regimen, tumor stage, age, or the time interval between imaging and therapy.

The analysis did not address MRI acquisition heterogeneity in detail. The pipeline did not include explicit harmonization for the scanner vendor, field strength, acquisition protocol, or temporal resolution of contrast-enhanced imaging. Radiomic features also depend on discretization, bin width, and resampling choices. We followed the feature set distributed with the public dataset, but did not perform a formal radiomic stability analysis.

The lesion ROI came from the public dataset segmentation. We did not assess the interobserver segmentation variability or its propagation to radiomic features and gating signals. The slice-window sensitivity analysis tested only three, five, and seven adjacent slices, without comparison to full 3D modeling or adaptive slice selection. Conventional clinical predictors and radiologist assessment were also outside the present comparison, so the incremental clinical value of the imaging model remains unknown.

Given these constraints, the study should be interpreted as a feasibility evaluation on one retrospective cohort. The present analysis also did not include formal statistical significance testing, PR-AUC reporting, calibration curves, decision-curve analysis, or model-efficiency evaluation. Larger multi-center cohorts, more diverse missingness patterns, clinical-variable integration, and prospective validation are needed before clinical use. Future work should also assess calibration and operating-threshold stability for the reliable identification of pCR-positive cases.

## 5. Conclusions

RGM-Net combines a multi-branch 2.5D encoder, radiomics-guided gating, and masked token fusion for pCR prediction from multi-sequence breast MRI under fixed sequence-availability settings. On the I-SPY1 Trial cohort, patient-level five-fold cross-validation showed higher fold-mean performance than channel-concatenation baselines built from the same imaging sequences. The five-slice 2.5D input achieved the highest mean AUC among the tested slice-window settings. The study remains limited to a single retrospective cohort and the fixed sequence combinations tested here. Independent multi-center validation, broader missingness evaluation, and the integration of clinical and molecular information are needed before clinical use.

## Figures and Tables

**Table 1:** Patient-level comparison of different methods under five-fold cross-validation. Deep-learning baseline rows use channel-concatenated DCE, T1-weighted, and T2-weighted inputs without random sequence dropping. The radiomics-only baseline is logistic regression on the 370 CaPTk features. Values are means ± standard deviations across folds (%, except AUC, which is on the [0,1] scale).

Method	Acc.	Sen.	Spe.	Pre.	F1	AUC
Radiomics-only (LR)	71.3 ± 2.1	55.8 ± 4.4	77.2 ± 1.9	48.0 ± 2.8	51.6 ± 3.2	0.711 ± 0.043
ResNet	72.6 ± 1.1	58.3 ± 3.8	78.1 ± 0.4	50.0 ± 0.0	53.8 ± 1.6	0.709 ± 0.074
ViT	73.9 ± 2.1	60.6 ± 4.9	79.0 ± 1.6	52.0 ± 2.8	55.9 ± 3.4	0.719 ± 0.049
EffNetV2	74.5 ± 1.7	62.8 ± 4.5	79.0 ± 1.6	52.9 ± 2.7	57.4 ± 3.2	0.747 ± 0.061
ConvNeXt	75.8 ± 1.2	67.5 ± 4.6	79.0 ± 1.6	54.7 ± 0.5	60.4 ± 1.6	0.788 ± 0.045
Proposed (RGG + MTF)	78.4 ± 2.2	70.0 ± 4.6	81.6 ± 1.9	58.9 ± 2.4	63.9 ± 2.8	0.809 ± 0.012

**Table 2:** Ablation study of the proposed components under patient-level five-fold cross-validation. Values are means ± standard deviations across folds.

Configuration	Acc.	Sen.	Spe.	Pre.	F1	AUC
ConvNeXt baseline	75.8 ± 1.2	67.5 ± 4.6	79.0 ± 1.6	54.7 ± 0.5	60.4 ± 1.6	0.788 ± 0.045
+ MTF only	76.5 ± 2.5	67.5 ± 4.6	79.8 ± 2.2	55.8 ± 4.2	61.1 ± 4.1	0.771 ± 0.031
+ RGG only	76.5 ± 2.2	70.0 ± 4.6	79.0 ± 1.6	55.6 ± 2.4	62.0 ± 3.0	0.765 ± 0.051
Full (RGG + MTF)	78.4 ± 2.2	70.0 ± 4.6	81.6 ± 1.9	58.9 ± 2.4	63.9 ± 2.8	0.809 ± 0.012

**Table 3:** Performance of the proposed framework under different sequence-availability settings (patient-level five-fold cross-validation). Values are means ± standard deviations across folds.

Available Sequences	Acc.	Sen.	Spe.	Pre.	F1	AUC
T1 + T2	73.9 ± 2.1	60.6 ± 4.9	79.0 ± 1.6	52.0 ± 2.8	55.9 ± 3.4	0.719 ± 0.044
DCE + T2	75.2 ± 2.4	62.8 ± 4.5	79.8 ± 2.2	54.0 ± 4.2	58.0 ± 4.1	0.722 ± 0.079
DCE + T1	76.4 ± 1.4	67.5 ± 4.6	79.8 ± 2.2	55.8 ± 2.4	61.0 ± 2.0	0.800 ± 0.050
All three sequences	78.4 ± 2.2	70.0 ± 4.6	81.6 ± 1.9	58.9 ± 2.4	63.9 ± 2.8	0.809 ± 0.012

**Table 4:** Performance of the proposed framework with different numbers of adjacent slices per 2.5D input (patient-level five-fold cross-validation). Values are means ± standard deviations across folds.

Slice Window	Acc.	Sen.	Spe.	Pre.	F1	AUC
3 adjacent slices	76.4 ± 1.4	67.5 ± 4.6	79.8 ± 2.2	55.8 ± 2.4	61.0 ± 2.0	0.763 ± 0.056
5 adjacent slices	78.4 ± 2.2	70.0 ± 4.6	81.6 ± 1.9	58.9 ± 2.4	63.9 ± 2.8	0.809 ± 0.012
7 adjacent slices	78.4 ± 2.2	72.2 ± 5.2	80.7 ± 2.3	58.6 ± 2.4	64.6 ± 3.0	0.799 ± 0.088

## Data Availability

The datasets analyzed during the current study are publicly available from The Cancer Imaging Archive (TCIA). The original I-SPY1 collection is available at https://www.cancerimagingarchive.net/collection/ispy1/ (accessed on 7 December 2025), and the ISPY1-Tumor-SEG-Radiomics analysis-result dataset is available at https://www.cancerimagingarchive.net/analysis-result/ispy1-tumor-seg-radiomics/ (accessed on 11 December 2025).
